# The heat shock protein 20 gene editing suppresses mycelial growth of *Botryosphaeria dothidea* and decreases its pathogenicity to postharvest apple fruits

**DOI:** 10.3389/fmicb.2022.930012

**Published:** 2022-07-27

**Authors:** Yonghong Huang, Junping Liu, Jinghui Li, Meng Sun, Yanxin Duan

**Affiliations:** ^1^College of Horticulture, Qingdao Agricultural University, Qingdao, China; ^2^Laboratory of Quality and Safety Risk Assessment for Fruit (Qingdao), Ministry of Agriculture and Rural Affairs, Qingdao, China; ^3^National Technology Centre for Whole Process Quality Control of FSEN Horticultural Products (Qingdao), Qingdao, China; ^4^Qingdao Key Laboratory of Modern Agriculture Quality and Safety Engineering, Qingdao, China

**Keywords:** *Botryosphaeria dothidea*, dimethyl trisulfide, Hsp20 gene family, CRISPR/Cas9, apple ring rot

## Abstract

Apple ring rot caused by *Botryosphaeria dothidea* is an essential and prevalent disease in the apple orchard in China. Our previous study demonstrated that dimethyl trisulfide (DT) from Chinese leek (*Allium tuberosum*) significantly suppressed the mycelial growth of *B. dothidea* and inhibited the incidence of apple ring rot postharvest. However, the mechanism underlying the inhibitory role of DT against *B. dothidea* is not fully understood. Comparing the control and the DT-treated *B. dothidea* mycelial transcriptomes revealed that heat shock protein 20 (Hsp20) strongly responded to DT treatment. This study identified four Hsp20 genes throughout the *B. dothidea* genome (BdHsp20_1-4). Each BdHsp20 gene had a conserved ACD with a variable N-terminal region and a short C-terminal extension. The segmental duplication event has contributed to the expansion of the BdHsp20 gene family. Compared to the wild-type strain, the CRISPR/Cas9 gene-edited BdHsp20 mutant (ΔBdHsp20) decreased the mycelial growth by 55.95% and reduced the disease symptom in postharvest apple fruit by 96.34%. However, the BdHsp20 complemented strain (ΔBdHsp20_C) significantly restored the growth and pathogenicity, which suggested that the BdHsp20 gene was closely involved in the growth and pathogenicity of *B. dothidea*. This study would accelerate the exploration of the molecular mechanism of the inhibitory effect of DT against *B. dothidea* and also provide new insights for the management of apple ring rot disease.

## Introduction

Apples are among the most important fruits globally, ranking fourth in world fruit production (Li et al., 2018). As of 2019, the Chinese apple area accounted for 43.27% (2.04 million hectares) of the total harvested area globally and reached 42.42 billion kilograms, accounting for 48.50% of world apple production (FAOSTAT, 2021). As a result, apple production has become a pillar industry in many rural areas in China. However, apple cultivation is threatened by apple ring rot caused by *Botryosphaeria dothidea*, bringing about fruit rot, stem and branch canker, and even tree death. Moreover, *B. dothidea* also infects many other fruit trees, including avocado (Qiu et al., 2020), mulberry (Huang et al., 2019), pomegranate (Gu et al., 2020), fig (Wang et al., 2020), sweet cherry (Zhang et al., 2019), kiwifruit (Wang et al., 2021a), and olive (Korukmez et al., 2019). The disease has become one of the most destructive diseases in China (Dong and Guo, 2020). Therefore, developing more effective measures to prevent and control the disease is crucial.

Until now, a synthetic fungicide is still the principal method of managing apple ring rot disease (Fan et al., 2019). However, excessive pesticide application causes fungicide resistance, environmental pollution, and public health concerns (Chen et al., 2019). In addition, it also results in phytotoxicity, increases cell membrane permeability, impairs metabolism, and declines quality and yield (Li et al., 2020b). Biological control offers a safer, environmental-friendly alternative without these shortcomings. In recent years, botanical pesticides and their derivatives have attracted extensive attention due to their effective control of various plant pathogens with minimal or no side effects (Chen et al., 2016; Singh et al., 2016). For example, the *Duranta repens* aqueous extract (durantol) exhibits promising inhibition against sorghum downy mildew (*Peronosclerospora sorghi*) (Singh et al., 2016). The pomegranate peel aqueous extract (punicalagins and ellagic acid) shows antifungal activity on Fusarium wilt of tomato caused by *Fusarium oxysporum* f. sp. *lycopersici* (Rongai et al., 2016). The *Sapindus mukorossi* extract (saponins) shows significant suppression against *Venturia inaequalis* and *Botrytis cinerea* (Porsche et al., 2018). A natural product cinnamic acid suppresses the mycelial growth of *Sclerotinia sclerotiorum*, and provides over 95% efficacy against *S. sclerotiorum* (Wang et al., 2019). Defensin from *Picea asperata* exhibits strong antifungal activity on *Pestalotiopsis neolitseae* (Liu et al., 2021). The cinnamon powder reduces the disease incidence of gray mold caused by *Botrytis cinerea* on tomato plants (Kowalska et al., 2019). Wheat multidomain cystatin TaMDC1 is involved in resistance against *Pseudomonas syringae, Botrytis cinerea*, and *Alternaria alternata* (Christova et al., 2018). Protein fractions IF25 obtained from *Solanum tuberosum* tubers show antifungal activity against two citrus-pathogenic fungi, *Penicillium digitatum* and *Geotrichum candidum* (Rodríguez et al., 2015). Therefore, natural fungicides have become a trend of pesticide development because they are effective, environment-friendly, readily biodegradable, and inexpensive (Swathi et al., 2010).

Hsp20 is a kind of heat shock protein with an average molecular weight of 20 kDa (Groenen et al., 1994), which is also called small Hsp (sHsp). sHSPs have chaperone-like activity *in vitro* and protect organisms from various stresses (Li et al., 2012). For example, Hsp20 in humans regulates apoptosis in hepatocellular carcinoma cells (Nagasawa et al., 2014) and might be a possible target for colorectal cancer therapy (Ju et al., 2015). Furthermore, Hsp20 plays an essential role in the innate immunity of pearl oyster *Pinctada martensii* (Lei et al., 2016), red swamp crayfish, *Procambarus clarkii* (Li et al., 2020a), and Japanese flounder (Yan et al., 2020). In plants, hsp20 confers heat and salt tolerance in rice (Guo et al., 2020), results in hypersensitivity to ABA in Arabidopsis (Yao et al., 2020; He et al., 2021), and responds to pathogen infection in tomatoes (Yu et al., 2016) and barley (Li and Liu, 2019). In the microbiome, Hsp20 is related to the heat tolerance of *Scrippsiella trochoidea* (Deng et al., 2020) and *Coriolopsis trogii* (Wang et al., 2021b), enhances tolerance to hydrogen peroxide stress of *Escherichia coli* (Singh et al., 2014), and is essential for the resistance to desiccation of *Azotobacter vinelandii* (Cocotl-Yanez et al., 2014).

A previous study found that dimethyl trisulfide (DT), one of the main components identified in Chinese leek (*Allium tuberosum*), completely suppressed the mycelial growth of *B. dothidea* and showed 97% inhibition against apple ring rot postharvest (Sun et al., 2022). Furthermore, transcriptome analysis revealed that the Hsp20 gene in *B. dothidea* (BdHsp20) highly responded to DT. Therefore, we deduced that the BdHsp20 gene was involved in the mycelial growth and the pathogenicity of *B. dothidea*. The study identified the BdHsp20 gene family throughout the *B. dothidea* genome. Furthermore, we investigated the effect of the BdHsp20 gene on the growth and pathogenicity of *B. dothidea* using the CRISPR/Cas9-based gene-editing method to explore the molecular mechanism underlying the inhibitory effect of DT against *B. dothidea*.

## Materials and methods

### Experimental materials

The apple fruit (*Malus domestica* Borkh. cv. Red Fuji) used in the experiments was purchased from local supermarkets. The fruit with uniform sizes, no disease spots, and no mechanical damage was selected for the experiments. DT was provided by the Micxy Reagent (Chengdu, China). The pathogen *B. dothidea* isolate Lw-1801 was kept on the potato dextrose agar (PDA) medium in the laboratory.

### Identification and basic feature analysis of the BdHsp20 gene family

First, the genomes of *B. dothidea* (GCA_011503125.2), *Diplodia seriata* (GCA_001975905.1), *Lasiodiplodia theobromae* (GCF_012971845.1), *Parastagonospora nodorum* (GCF_000146915.1), *Zymoseptoria tritici* (GCF_000219625.1), and *Valsa mali* (GCA_000818155.1) were downloaded from the National Center for Biotechnology Information (NCBI) genome website (https://www.ncbi.nlm.nih.gov/). In addition, the Hsp20 protein Pfam model (PF00011) was downloaded from the Pfam database (http://pfam.xfam.org/). Then, using HMMER software, the Hsp20 genes were identified across the six genomes. Finally, the identified Hsp20 genes were confirmed with the NCBI Conserved Domain Database (CDD) and Simple Modular Architecture Research Tool database (SAMRT) (http://smart.embl-heidelberg.de/l).

The molecular weight (MW) and isoelectric point (PI) of the BdHsp20 gene family were calculated by online software Expasy (https://web.expasy.org/compute_pi/). The motif of the BdHsp20 gene family was analyzed using the MEME website (http://meme-suite.org/tools/meme). ClustalW was used to align the sequence of the BdHsp20 gene family, and then a phylogenetic tree was generated by using the maximum likelihood (ML) method with 1,000 bootstrap replicates using the MEGA X program. The collinearity was analyzed using MCScanX. The subcellular localization was predicted using the online software ProtComp9.0 (http://linux1.softberry.com.berry.phtml). Finally, the phylogenetic tree, conserved domain, gene structure, gene motif, and collinearity were visualized by Tbtools software (Chen et al., 2020).

### The prediction of promoter and transcription factor-binding sites of the BdHsp20 gene family

The 2,000-bp sequences upstream of the four BdHsp20 genes were extracted from the *B. dothidea* genome (GCA_011503125.2) to analyze the promotors and transcription factor-binding sites. Online tools Promoter 2.0 (https://services.healthtech.dtu.dk/service.php?Promoter-2.0) and Neural Network Promoter Prediction (https://fruitfly.org/seq_tools/promoter.html) were used to predict the promoter regions of the four BdHsp20 genes. The transcription factor-binding sites were predicted using the JASPAR CORE database (https://jaspar.genereg.net/) with default parameters, except a relative profile score threshold of 100%.

### The BdHsp20 gene expression pattern induced by dimethyl trisulfide

A mycelia disc (0.5 cm in diameter) of *B. dothidea* was inoculated at the center of a layer of cellophane laid over the PDA medium (20 mL) contained in a Petri dish (70 mL in volume). The Petri dish was inverted and incubated at 28°C in the dark for 48 h. Then 100 μL of DT (125 ml/L) was added to a small piece of sterilized filter paper (2 × 2 cm) that was placed on the inner lid of the Petri dish. A measure of 100 μL of sterilized water was used as the control. After that, the mycelia were continued to be incubated under the same condition. Finally, the mycelia were collected 1 h, 6 h, and 12 h later to determine the BdHsp20 gene expression.

The FPKM values of BdHsp20 genes treated with DT were downloaded from the NCBI (PRJNA727720). Then, the qRT-PCR analysis was performed to verify the relative expression of the BdHsp20 gene once more. First, the sequence information of the BdHsp20 genes was extracted from the *B. dothidea* genome, and the primers were designed using NCBI Primer-BLAST (https://www.ncbi.nlm.nih.gov/tools/primer-blast/) (Supplementary Table 1). Then, the cDNA was synthesized using the HiScript^®^ lll RT SuperMix for qPCR(+gDNA wiper)reverse transcription kit (Vazyme Biotech, Nanjing, CN). According to the ChamQTM SYBR Color qPCR Q25 Master Mix kit (Vazyme Biotech, Nanjing, CN), the expression of BdHsp20 genes in B. dothidea treated with DT was analyzed by using an ABI7500 thermal cycler (Applied Biosystems, CA). The total reaction system was 10 μl, including 5 μL of 2 × ChamQ SYBR Color qPCR Master Mix, 0.2 μL of each primer, 0.2 μL of the 50 × ROX Reference DyeI, 1 μL of cDNA, and 3.4 μL of the ddH_2_O. The reaction conditions were as follows: 94°C for 5 min, followed by 30 cycles of 94°C for 30 s and 60°C for 30 s, then 72 °C for 30 s, and a final extension at 72°C for 10 min. Actin was used as the internal reference gene, and the relative expression was calculated using the 2^−Δ*ΔCT*^ method (Livak and Schmittgen, 2001). Three biological replicates were set up in the experiment.

### Preparation of the BdHsp20 gene-edited and the complemented *Botryosphaeria dothidea*

For the BdHsp20 gene-edited mutant, we submitted the BdHsp20_1 gene (GTA08_BOTSDO12492.1) sequence to CRISPOR (http://crispor.tefor.net/) website to design the sgRNA. First, according to the guide sequence ACTCCCTGGCATTGCTCAGA*AGG*, one pair of primers, 5'- *CACCG*ACTCCCTGGCATTGCTCAGA-3', 5'- *AAAC*TCTGAGCAATGCCAGGGAGT*C*-3', was designed. Next, the vector plasmid PX458 was digested with restriction endonuclease *BbsI* and ligated with the double-stranded gRNA by the T4 DNA ligase, thus forming the recombinant plasmid PX458-BdHsp20 (Supplementary Figures 1A,B). Then the recombinant plasmid PX458-BdHsp20 was introduced into the *B. dothidea* protoplast using the polyethylene glycol (PEG)-mediated transformation method (Hagiwara et al., 2013). Subsequently, the mixture was spread onto a PDA medium to screen the transformant. Finally, the apical mycelia from a single colony were picked and inoculated on the medium for a second screening. This screening process was repeated at least three times. After that, the BdHsp20 gene was amplified from the transformed strain using the primers: F: 5'- ATGTCGATGTTCCCGCG-3', R: 5'- TTACTCGATGTTGATCCTGCGA-3', and the PCR product was sequenced to verify the gene-edit site. The final gene-edited *B. dothidea* mutant was designated as ΔBdHsp20.

For the BdHsp20 gene complemented *B. dothidea*, first, the *B. dothidea* mycelia RNA was extracted using the RNAprep Pure Plant Plus Kit (Tiangen Biotech (Beijing) Co., Ltd, Beijing, China), and cDNA was synthesized by HiScript^®^ III RT SuperMix for qPCR (+gDNA wiper) (Nanjing Vazyme Biological Technology Co., Ltd, China). Then, the synthesized cDNA was used as a template to obtain the full-length coding sequences (CDS) of BdHsp20 with the following primers: F: 5'-ttgatacatatgcccgtcgacATGTCGATGTTCCCGCG-3', R: 5'-ccttgctcaccatggatccTTACTCGATGTTGATCCTGCG-3'. Next, the expression vector PRI101 was double-digested with restriction enzymes *BamH* I and *Sal* I, followed by ligation with the previously amplified BdHsp20 CDS using a CloneExpression^®^ MultiS One Step Cloning Kit (Vazyme Biotech, Nanjing, CN). The recombinant plasmid was designated as PRI101-BdHsp20 (Supplementary Figures 1C,D), which was transformed into the ΔBdHsp20 protoplast, respectively, using the polyethylene glycol (PEG)-mediated transformation method (Hagiwara et al., 2013). Finally, the mixture was spread onto a PDA medium to screen the transformant. The apical mycelia from a single colony were picked and inoculated on the medium for a second screening. This screening process was repeated at least three times. The BdHsp20 gene was amplified from the transformed strain with the primers: F: 5'-TATCCTTCGCAAGACCCTTC-3', R: 5'- TTACTCGATGTTGATCCTGCGA-3'. Then, the PCR product was sequenced to verify the gene complement, and the final complemented strain was designated as ΔBdHsp20_C.

### The effect of the BdHsp20 gene on the *Botryosphaeria dothidea* growth

A volume of 20 ml of PDA medium was poured into a Petri dish (9 cm in diameter). A mycelial disc (0.5 cm in diameter) of ΔBdHsp20 and ΔBdHsp20_C strain was inoculated onto the center of the PDA medium, respectively. A mycelial disc of the wild-type strain was used as a control. All the Petri dishes were inverted and incubated at 28°C in the dark for 3 days. The colony diameters of the three *B. dothidea* strains were measured to evaluate the effect of the BdHsp20 gene on the mycelia growth of *B. dothidea*. The experiments were repeated 14 times for each treatment.

### The effect of the BdHsp20 gene on the *Botryosphaeria dothidea* pathogenicity

Apple fruit with uniform sizes, free of mechanical damage or visible disease, was selected as the material. The fruit was surface-sterilized with 1% sodium hypochlorite for 10 min, washed three times with sterilized water, and then air-dried. A minor wound (3 mm wide, 3 mm deep) was made at the equator of the apple fruit with a sterile puncher. A mycelial disc (3 cm in diameter) of the ΔBdHsp20 and ΔBdHsp20_C strain was inoculated into the wound. A mycelial disc of wild-type *B. dothidea* strain was used as a control. All the fruits were incubated at 28°C in the dark for 5 days. The diameter of the disease spots on apple fruits was measured to evaluate the pathogenicity of the strains. The experiments were repeated 20 times for each treatment.

## Results

### The identification and the basic features of the BdHsp20 gene family

In the *B. dothidea* genome, five genes (GTA08_BOTSDO02788.1, GTA08_BOTSDO12492.1, GTA08_BOTSDO08183.1, GTA08_BOTSDO09929.1, and GTA08_BOTSDO02651.1) were annotated as Hsp20. Using HMMER software, four candidate Hsp20 genes (GTA08_BOTSDO02788.1, GTA08_BOTSDO12492.1, GTA08_BOTSDO08183.1, and GTA08_BOTSDO09929.1) were searched from the *B. dothidea* genome. However, after being authenticated in the NCBI database, four genes, GTA08_BOTSDO02788.1, GTA08_BOTSDO12492.1, GTA08_BOTSDO08183.1, and GTA08_BOTSDO09929.1, were found to have a conserved α-crystallin domain (ACD)_sHsps-like domain, which was also confirmed through the SMART database. Therefore, the four genes were regarded as Hsp20 genes in *B. dothidea* (BdHsp20). They were designated as BdHsp20_1 - BdHsp20_4 according to their distribution order on the contigs. The length of the encoding proteins was between 181 and 318 amino acids, the predicted isoelectric point ranged from 5.49 to 6.37, and the molecular weight ranged from 20.75 to 34.63 kD. In addition, ProtComp9.0 prediction suggested that BdHsp20_1 and BdHsp20_2 were primarily localized in the cytoplasm, whereas BdHsp20_3 and BdHsp20_4 in the endoplasmic reticulum and mitochondria, respectively (Table 1), which meant each BdHsp20 might have different functions.

**Table 1:** Basic features of the BdHsp20 gene family.

**Gene name**	**Gene ID**	**Contig**	**Location**	**Length/aa**	**PI**	**MW/kD**	**SL**.
BdHsp20_1	GTA08_BOTSDO12492.1	7	1600387–1600958	181	5.49	20.75	Cy
BdHsp20_2	GTA08_BOTSDO02788.1	16	3299873–3300444	181	5.49	20.75	Cy
BdHsp20_3	GTA08_BOTSDO08183.1	51	2664231–2665187	318	5.65	34.63	ER
BdHsp20_4	GTA08_BOTSDO09929.1	73	1214781–1215539	252	6.37	27.65	Mt

*MW, molecular weight; SL, subcellular localization; Er, endoplasmic reticulum; Mt, mitochondria, Cy, cytoplasm*.

### The phylogenetic analysis of the Hsp20 gene family

**Figure 1:**
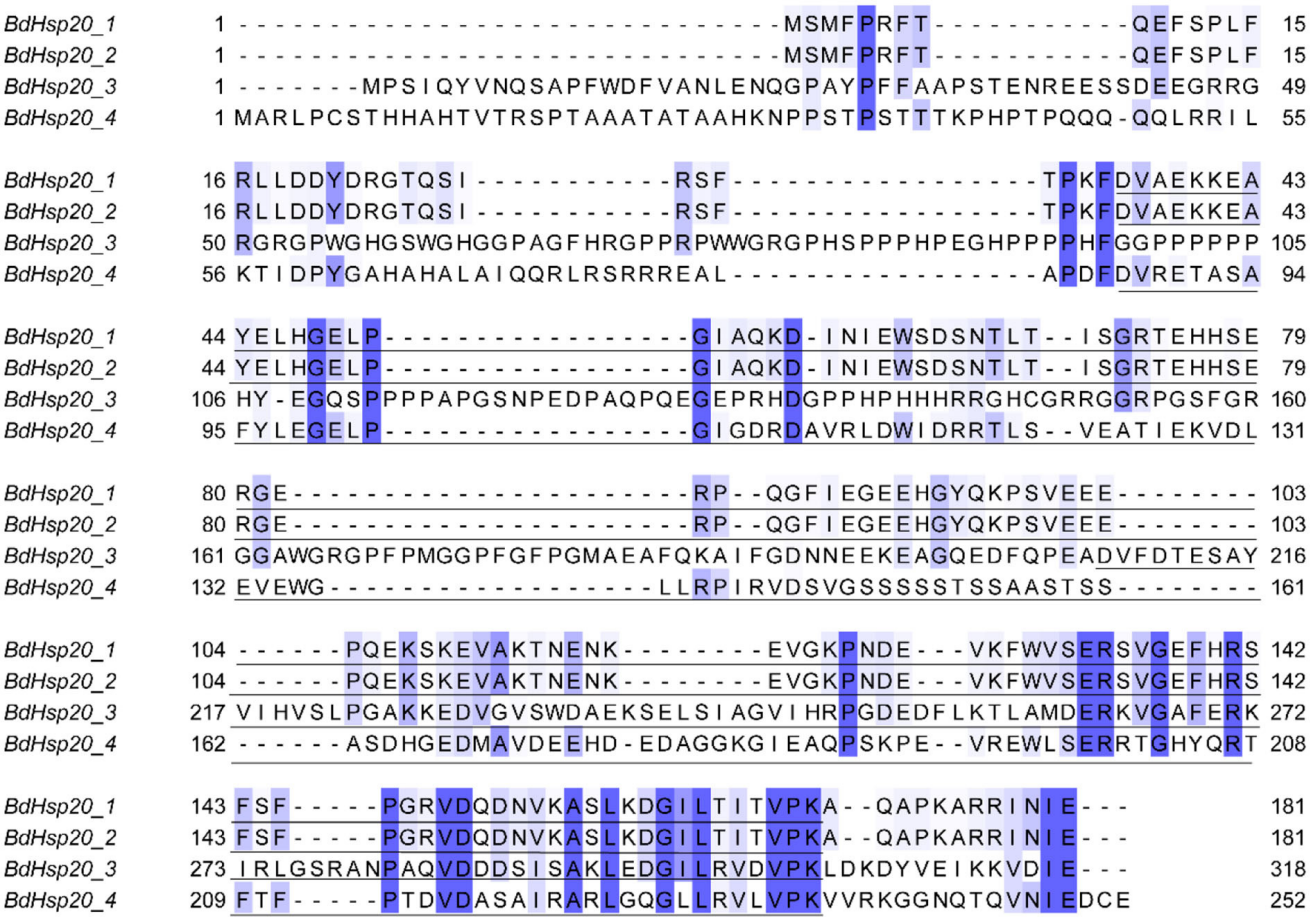
Alignment of the BdHsp20 gene family. The underlined sequences in BdHsp20_1 (36 aa−169 aa), BdHsp20_2 (36 aa−169 aa), BdHsp20_3 (208 aa−304 aa), and BdHsp20_4 (87 aa−235 aa), were the conserved alpha-crystallin domain. Each sequence also has a variable N-terminal region and a short C-terminal extension.

**Figure 2:**
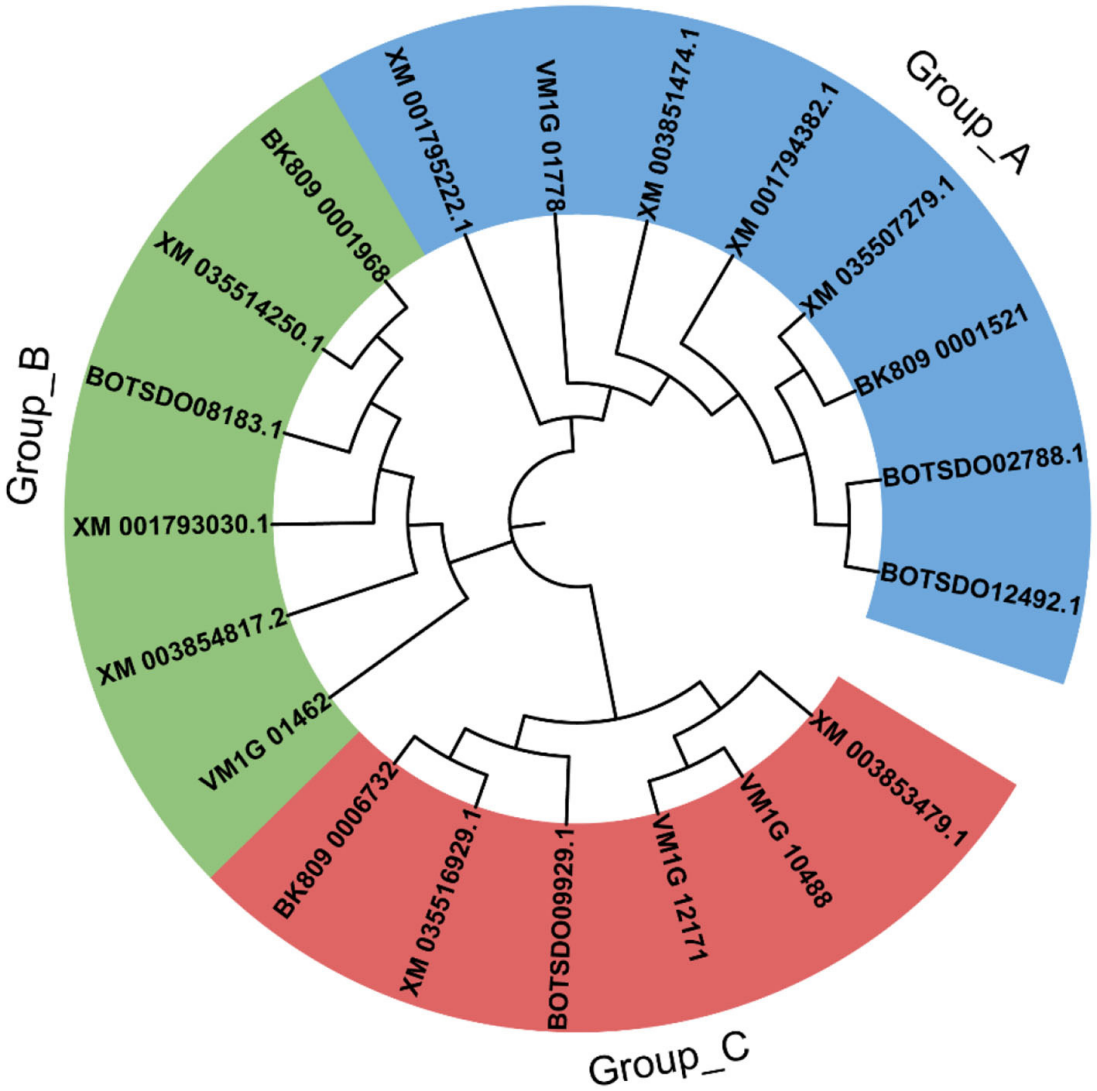
Phylogenetic tree constructed by the Hsp20 gene family of the six species, including *Botryosphaeria dothidea* (BdHsp20_2, BdHsp20_1, BdHsp20_3, BdHsp20_4), *Diplodia seriata* (BK809_0001521, BK809_0001968, BK809_0006732), *Lasiodiplodia theobromae* (XM_035507279.1, XM_035514250.1, XM_035516929.1), *Parastagonospora nodorum* (XM_001794382.1, XM_001795222.1, XM_001793030.1), *Zymoseptoria tritici* (XM_003851474.1, XM_003854817.2, XM_003853479.1), and *Valsa mali* (VM1G_10488, VM1G_01462, VM1G_12171, VM1G_01778).

The sequence identity of the four BdHsp20 genes was 35.14%. However, the sequence identity of BdHsp20_1 and BdHsp20_2 was as high as 100%. Each gene harbored a conserved α-crystallin domain (ACD)_sHsps-like domain with a variable N-terminal region and a short C-terminal extension (Figure 1). To further investigate the evolutionary relationship among fungal Hsp20 proteins, in addition to the four BdHsp20 genes, we identified other 16 Hsp20 genes throughout other five species, including *D. seriata* (3), *L. theobromae* (3), *P. nodorum* (3), *Z. tritici* (3), and *V. mali* (4). The phylogenetic analysis showed that the 20 Hsp20 genes were clustered into three groups. Group A comprised eight genes from six species, including BdHsp20_1 and BdHsp20_2; group B contained six genes from six species, including BdHsp20_4. Finally, group C involved six genes from five species. BdHsp20 genes showed a closer relationship in the three groups with the Hsp20 genes from *L. theobromae* and *D. seriata* (Figure 2), indicating that Hsp20 from the more closely related species had a closer phylogenetic relationship.

### The motif and gene structure of the BdHsp20 gene family

**Figure 3:**
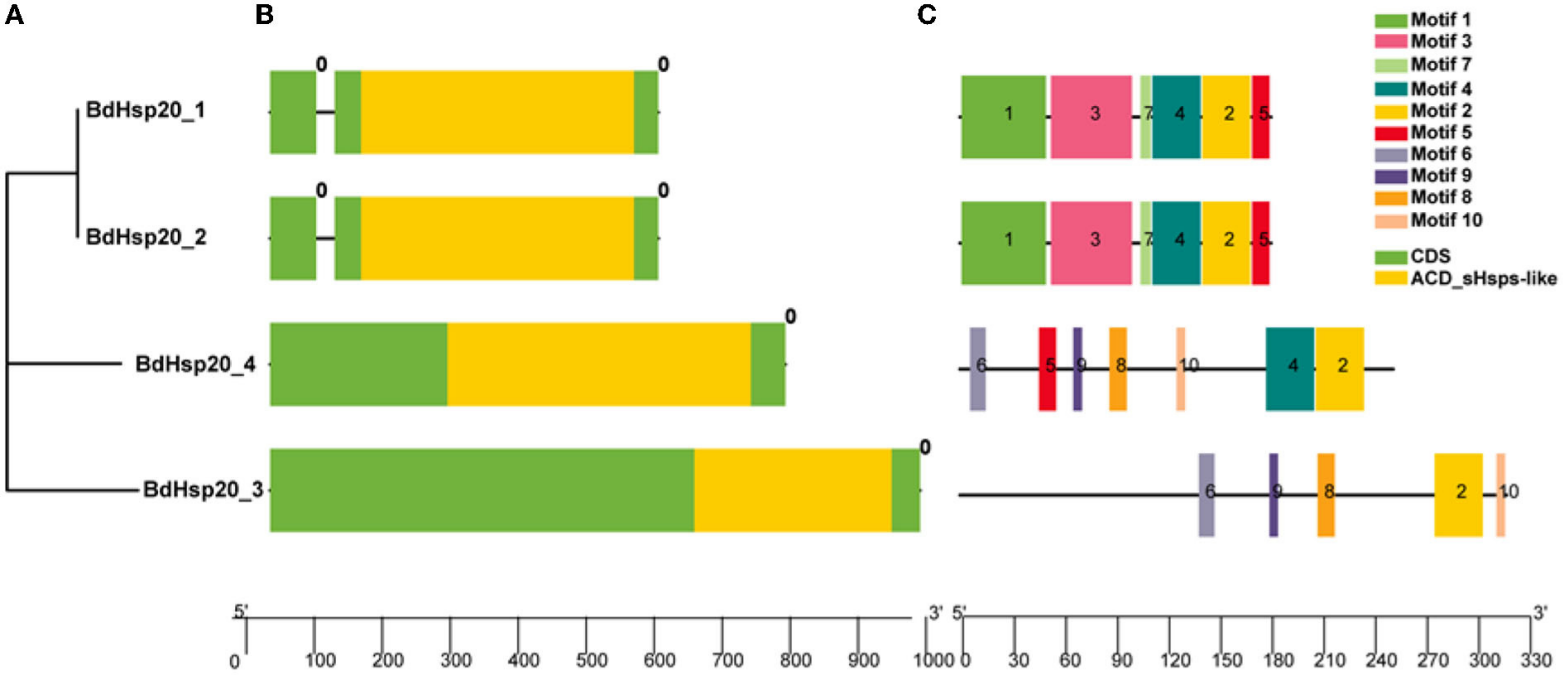
Phylogenetic relationships **(A)**, conserved domains, gene structures **(B)** and motifs **(C)** of the BdHsp20 gene family.

The phylogenetic analysis revealed that BdHsp20_1 and BdHsp20_2 were clustered into one group, while BdHsp20_3 and BdHsp20_4 were clustered into other two groups, respectively (Figure 3A). Each of the BdHsp20 gene contained a conserved domain ACD_sHsps-like at the C terminus, 96 (BdHsp20_3)-148 bp (BdHsp20_4) in length (Figure 3B). Gene structure analysis showed that BdHsp20_1 and BdHsp20_2 contained two exon regions (69 bp and 477 bp in length, respectively) and one intron region (26 bp in length). However, BdHsp20_3 and BdHsp20_4 consisted of only exons without any introns (Figure 3B). We also analyzed the motifs in the BdHsp20 gene family (Supplementary Table 2). BdHsp20_1 and BdHsp20_2 contained six common motifs, including motif 1, motif 2, motif 3, motif 4, motif 5, and motif 7. In addition, BdHsp20_3 and BdHsp20_4 also had five identical motifs such as motif 2, motif 6, motif 8, motif 9, and motif 10. Among the 10 identified motifs, motif 2 was the only common motif in the BdHsp20 gene family (Figure 3C). These analyses implied that the more closely related BhHsp20 genes have similar gene sequence features.

### BdHsp20 gene distribution and collinearity analysis

**Figure 4:**
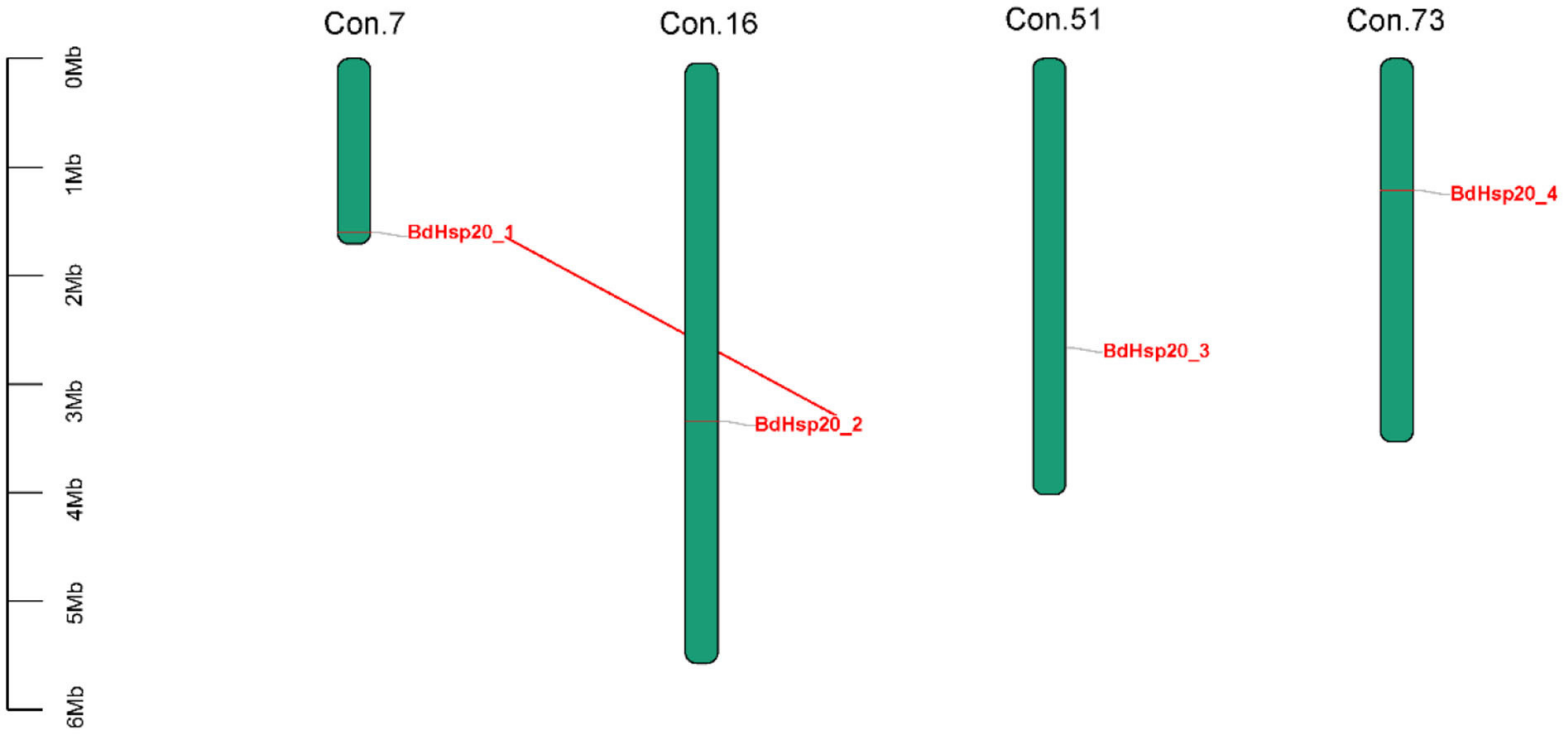
Distribution of BdHsp20 gene family members on the *Botryosphaeria dothidea* contigs. Two genes linked with a red line represent a paralogous gene pair.

**Figure 5:**
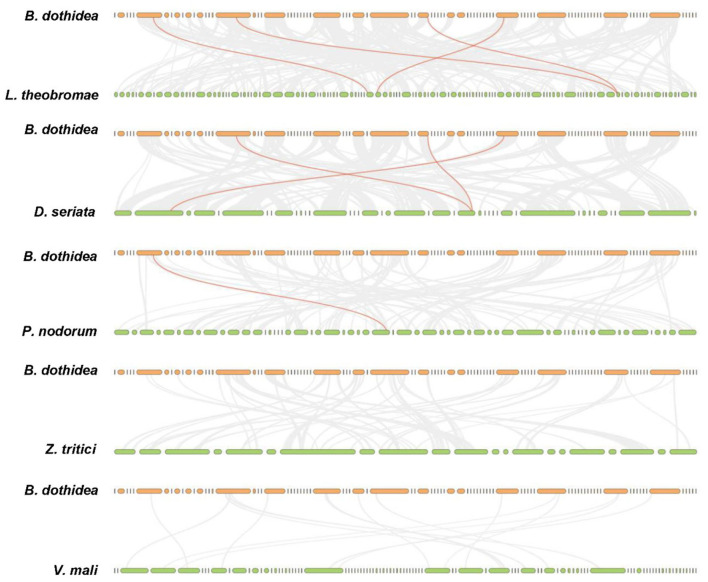
Collinearity analysis of Hsp20 genes between *Botryosphaeria dothidea* and five representative fungal species. Gray lines in the background indicate the collinear blocks within *Botryosphaeria dothidea* and other fungal genomes, while the red lines highlight the syntenic Hsp20 gene pairs. The species names with the prefixes '*D. seriata*', '*L. theobromae*', '*P. nodorum*', '*Z. tritici*', and '*V. mali*' indicate *Diplodia seriata, Lasiodiplodia theobromae, Parastagonospora nodorum, Zymoseptoria tritici*, and *Valsa mali*, respectively.

Totally, four BdHsp20 genes were distributed on the contig 7, contig 16, contig 51, and contig 73, respectively. The collinearity analysis revealed that BdHsp20_1 and BdHsp20_2 were clustered into a segmental duplication event, and they were one pair of paralogous genes (Figure 4), which indicated the segmental duplication event led to the expansion of the gene family. To further infer phylogenetic mechanisms of the BdHsp20 gene family, we constructed comparative collinearity maps of *B. dothidea* and the other five representative related species such as *L. theobromae, D. seriata, P. nodorum, Z. tritici*, and *V. mali* (Figure 5). From the map, four orthologous pairs, BdHsp20_1/XM_035507279.1 (Ka/Ks = 0.1697), BdHsp20_2/XM_035507279.1 (Ka/Ks = 0.1697), BdHsp20_3/XM_035514250.1 (Ka/Ks = 0.1593), and BdHsp20_4/XM_035516929.1 (Ka/Ks = 0.4659), were identified between *B. dothidea* and *L. theobromae*; three orthologous pairs, BdHsp20_1/BK809_0001521 (Ka/Ks=0.6998), BdHsp20_2/ BK809_0001521 (Ka/Ks= 0.6998), and BdHsp20_4/ BK809_0006732) (Ka/Ks= 0.7448), were identified between *B. dothidea* and *D. seriata*; and one orthologous pair, BdHsp20_3/XM_001793030.1 (Ka/Ks= 0.2706), was identified between *B. dothidea* and *P. nodorum*. The Ka/Ks of all the orthologous pairs were <1, suggesting that the BdHsp20 gene family might have experienced a strong purifying selective pressure during evolution.

### The prediction and the transcription factor-binding sites in the BdHsp20 gene family

**Figure 6:**
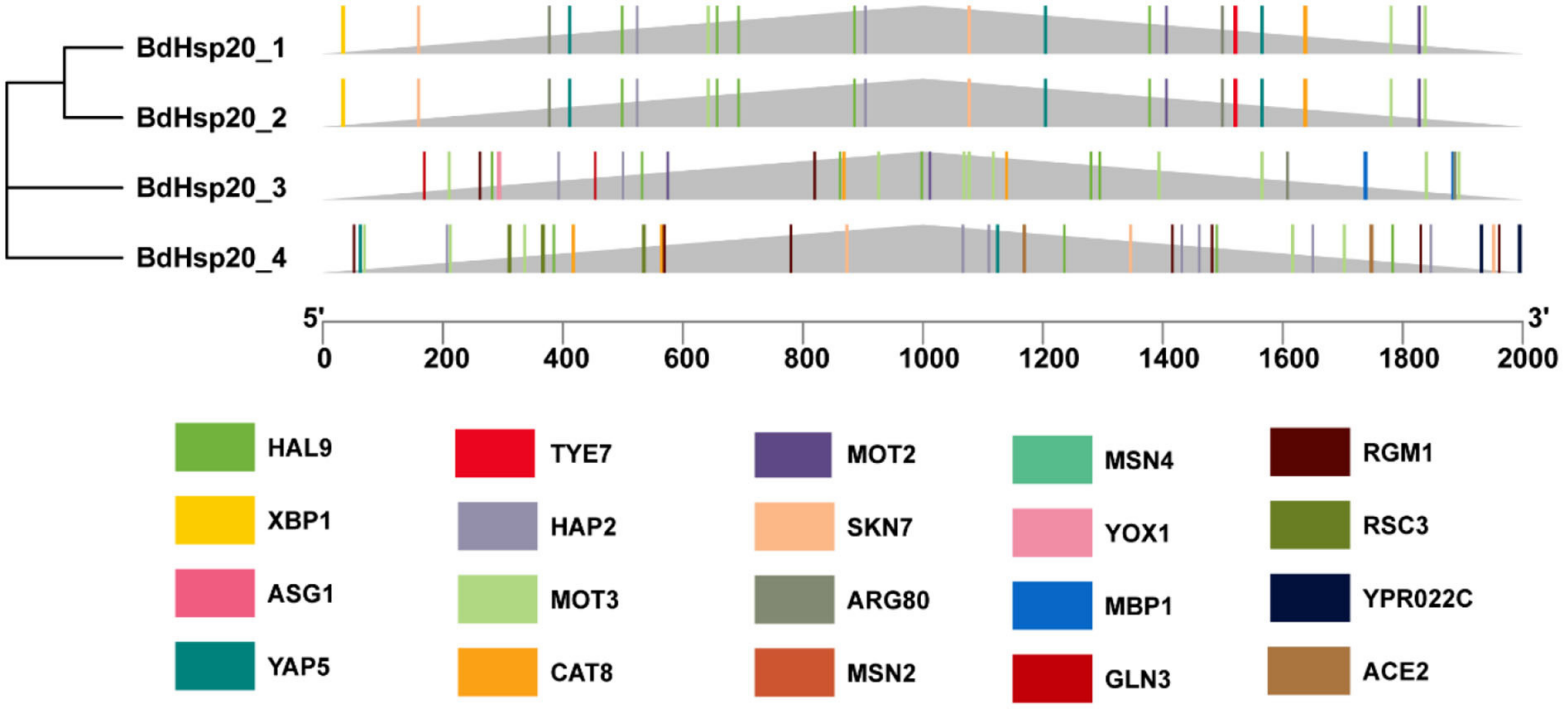
Schematic diagram of the transcription factor-binding sites of the BdHsp20 gene family predicted by using JASPAR online tool.

Promoter 2.0 analysis revealed that BdHsp20_1 and BdHsp20_2 contained a potential promoter, BdHsp20_3 had two promoters, and BdHsp20_4 had no promotor (Supplementary Table 3). NNPP analysis showed that BdHsp20_1, BdHsp20_2, and BdHsp20_3 contained three likely promoters, respectively, while BdHsp20_4 included five likely promoters (Supplementary Table 4). JASPAR revealed that BdHsp20_1 and BdHsp20_2 had the same transcription factor-binding sites, the number of which was 46, belonging to 27 categories such as HAL9 (7), YAP5 (3), MOT3 (3), and MOT2 (3). BdHsp20_3 had 53 transcription factor-binding sites, which belonged to 27 categories, including MOT3 (9), HAL9 (8), and MSN2 (2), while BdHsp20_4 contained 25 categories consisting of 67 transcription factor-binding sites such as MSN2 (7), MSN4 (7), HAP2 (7), and RGM1 (7). The four BdHsp20 genes shared 12 transcription factor-binding sites, including GLN3, ASG1, RGM1, CAT8, MSN2, MSN4, SKN7, MET28, MOT3, HAP2, YAP5, and HAL9 (Figure 6).

### Dimethyl trisulfide significantly induced the expression of the BdHsp20 gene family

**Figure 7:**
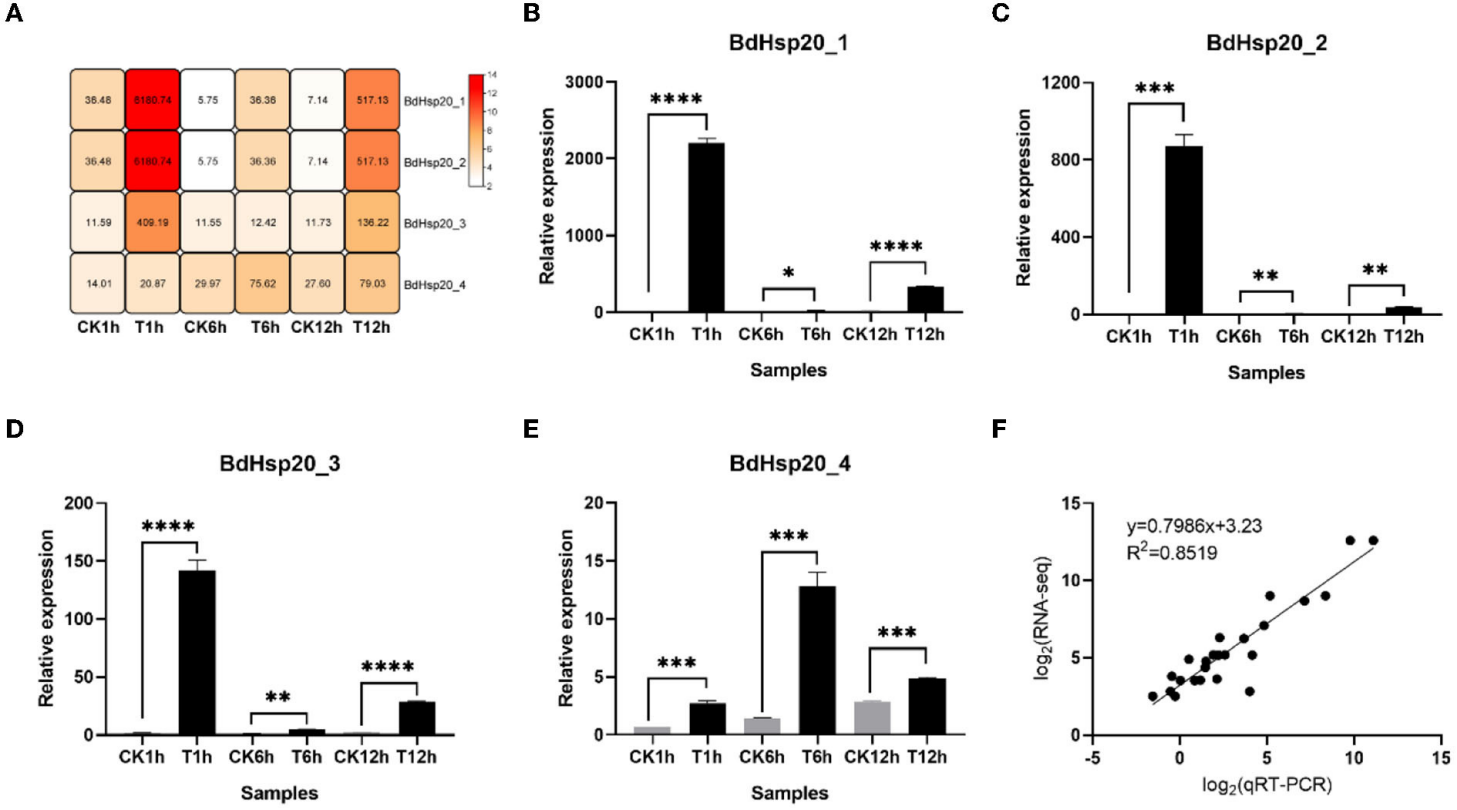
Heatmap of constructed by FPKM value of BdHsp20 genes assayed by RNA-seq **(A)**, the relative expression of BdHsp20 genes determined by qRT-PCR **(B–E)**, and correlation analysis of FPKM value and relative expression of BdHsp20 **(F)**. *P < 0.05, **P < 0.01, ***P < 0.001, ****P < 0.0001.

RNA-seq data showed DT significantly induced the BdHsp20 gene family throughout the experiment. Compared to the untreated control, the expressions of the four genes in the DT-treated mycelia were upregulated by 1.49–169.44-fold at 1 h, 1.07–6.32-fold at 6 h, and 2.87–72.47-fold at 12 h, respectively. In addition, the qRT-PCR analysis showed that the expressions of the BdHsp20 gene family in DT-treated fungal mycelia demonstrated 3.77–367.59-fold higher at 1 h, 4.28–21.37-fold higher at 6 h, and 1.72–52.62-fold higher at 12 h than that in the untreated control. The expression trends of qRT-PCR were consistent with the RNA-seq results, and they have a high correlation (R^2^ = 0.8519) (Figure 7), confirming the inductive effect of DT on the BdHsp20 gene family.

### BdHsp20 gene editing slowed down the growth of *Botryosphaeria dothidea*

**Figure 8:**
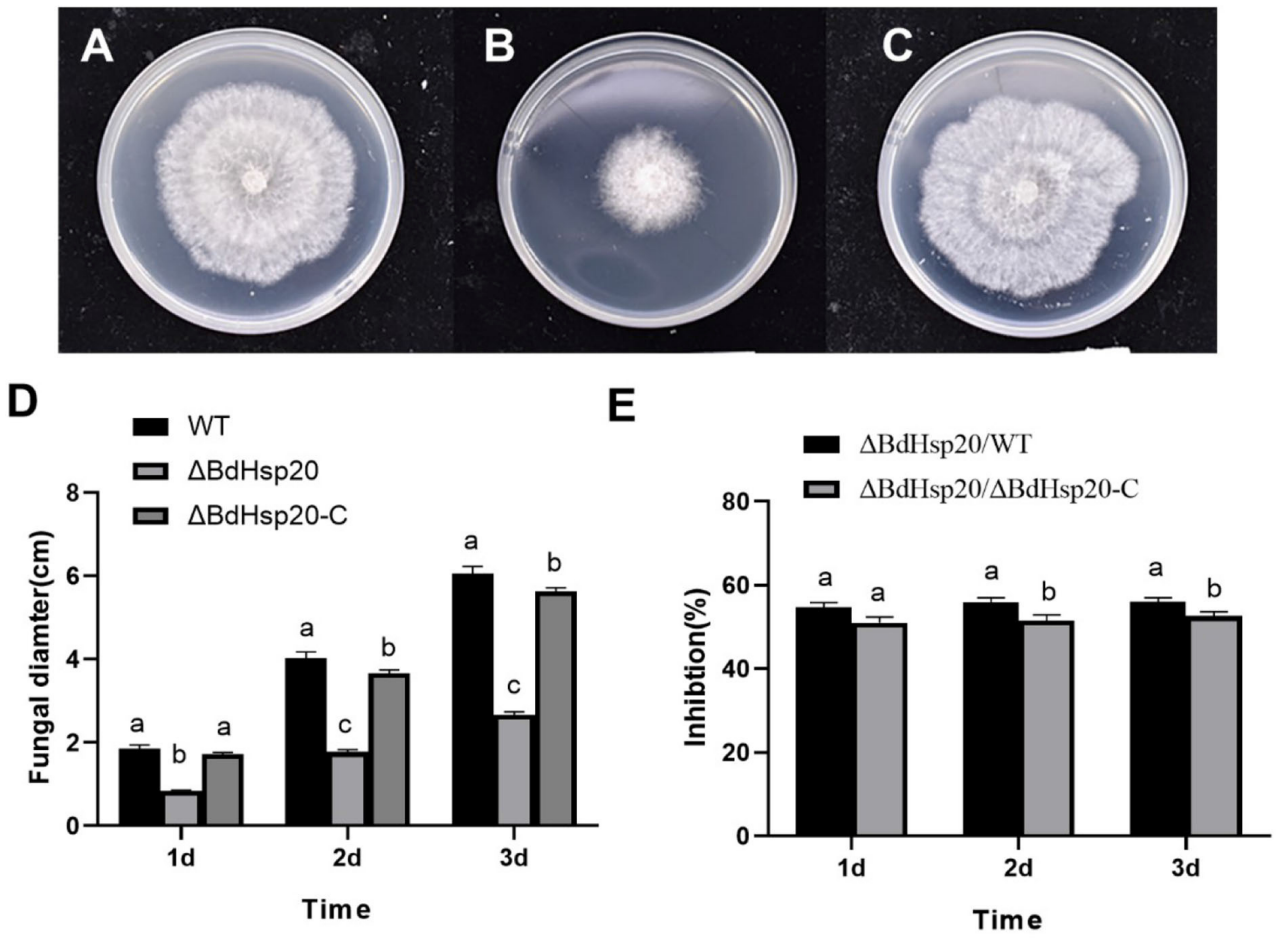
Mycelial growth of the three *Botryosphaeria dothidea* strains. The wild-type strain **(A)**, the CRISPR/Cas9 gene-edited BdHsp20 strain (ΔBdHsp20) **(B)**, and the BdHsp20 complemented strain (ΔBdHsp20_C) **(C)** were inoculated on a PDA medium for three days. The fungal colony diameters of the three strains were significantly different **(D)**. Compared to the wild type (ΔBdHsp20/WT) and the ΔBdHsp20_C (1BdHsp20/1BdHsp20_C), BdHsp20 gene deletion significantly inhibited the mycelial growth of *Botryosphaeria dothidea* **(E)**. Lowercase letters indicate significant differences between treatments or times (*P* < 0.05).

Sequence analysis revealed that compared to the wild-type strain, sgRNA-mediated editing of BdHsp20 triggered a single-base substitution and deletion at the target gene and its vicinity in the ΔBdHsp20 strain. We speculated that this deletion probably led to the premature termination of BdHsp20 protein translation, further resulting in the reduction of BdHsp20 protein from 181 amino acids to 67 amino acids, bringing about incomplete BdHsp20 protein and deletion of the conserved ACD (Supplementary Figure 2). Next, the wild-type, ΔBdHsp20, and ΔBdHsp20_C strains were inoculated on the PDA medium to test their growth. After 1 day, all three strains began to grow. But the mycelia of the wild-type strain and ΔBdHsp20_C strain grew faster than those of ΔBdHsp20 on the subsequent days. After 3 days, the mycelia of the wild-type strain and ΔBdHsp20_C strain nearly covered the whole surface of the medium. However, the ΔBdHsp20 strain is relatively small, covering less than half of the surface of the medium (Figures 8A–C). The statistics showed that the mycelial diameter of the ΔBdHsp20 strain was only 2.67 cm 3 days after inoculation (Figure 8D), which was reduced by 55.95% compared to the that of wild-type strain, also 52.54% smaller than that of the ΔBdHsp20_C strain (Figure 8E).

### BdHsp20 gene editing reduced the ring rot disease on the postharvest apple fruits

**Figure 9:**
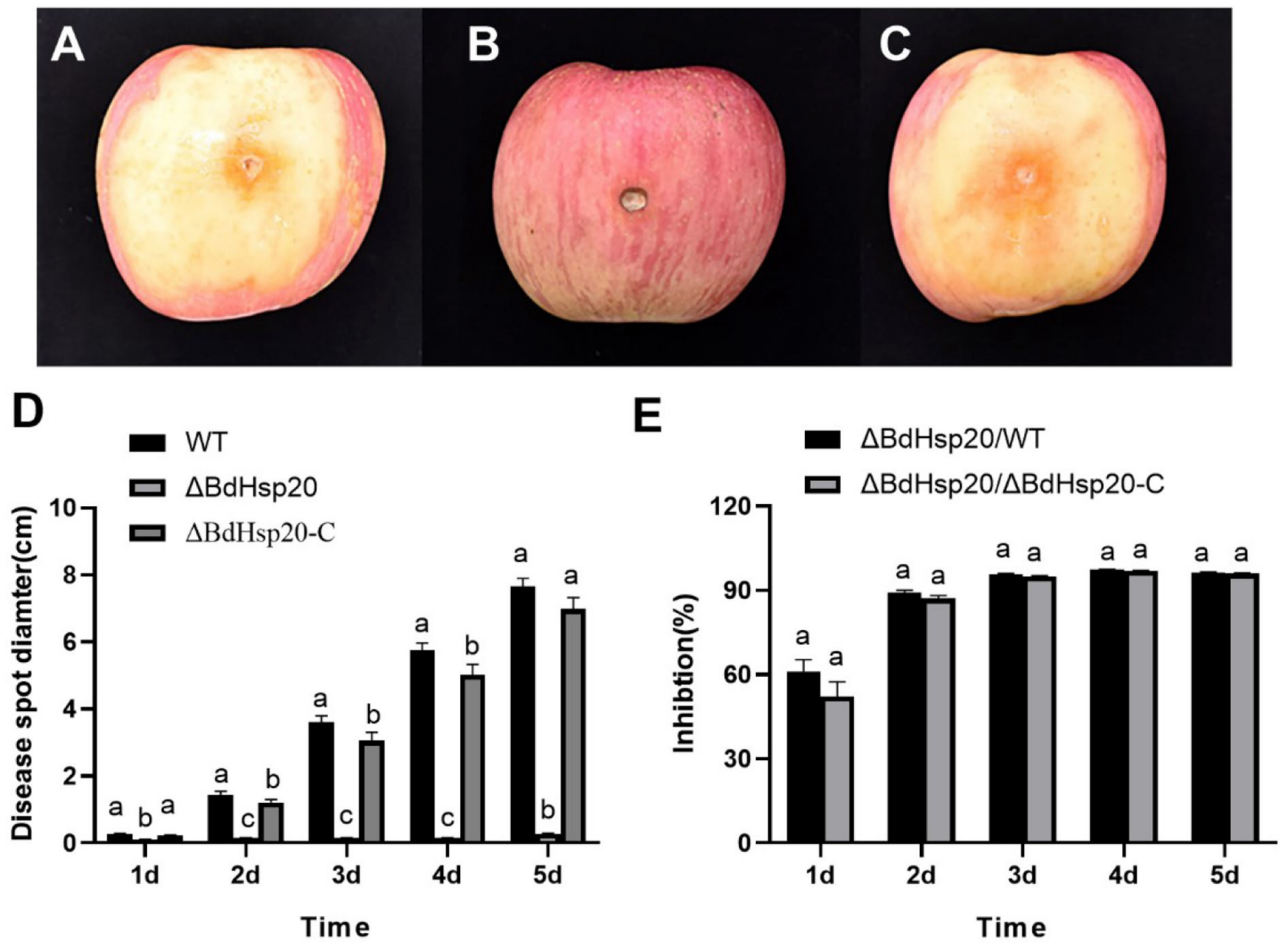
Pathogenicity of *Botryosphaeria dothidea*. The apple fruit inoculated with the wild-type strain **(A)**, the CRISPR/Cas9 gene-edited BdHsp20 strain (ΔBdHsp20) **(B)**, and the BdHsp20 complemented strain (ΔBdHsp20_C) **(C)** for 5 days. The disease spot diameter on apple fruit inoculated with the three strains was significantly different **(D)**. Compared to the wild type (ΔBdHsp20/WT) and ΔBdHsp20_C (ΔBdHsp20/ΔBdHsp20_C), BdHsp20 gene deletion significantly reduced the pathogenicity of *Botryosphaeria dothidea* on postharvest apple fruits **(E)**. Lowercase letters indicate significant differences between treatments or times (*P* < 0.05).

On the first day, the apple fruits inoculated with the mycelial discs of the wild-type strain and the ΔBdHsp20_C strain began to show disease symptoms at the inoculated point. After that, the disease spot gradually expanded in the subsequent days, and the rotten symptom slowly emerged on the tissue of the inoculation point. Finally, 5 days after inoculation, the disease spot extended to the whole surface of the apple fruits, and the tissue around the inoculation point was severely rotten with exudating mucus. But the apple fruits inoculated with the ΔBdHsp20 mycelial disc showed a slight disease symptom during the experiment period (Figures 9A–C). The statistics showed that the disease spot diameter of apple fruit inoculated with the wild-type strain and the ΔBdHsp20_C strain was significantly larger than that of the ΔBdHsp20 throughout the experiment time (Figure 9D). At 5 days after inoculation, the disease spot diameter on apple fruit inoculated with the ΔBdHsp20 strain was 0.28 cm, which was reduced by 96.34% compared to that of the wild-type strain and was also smaller by 95.99% than that of the ΔBdHsp20_C strain (Figure 9E).

## Discussion

In the present study, we identified four members of the Hsp20 gene family in the *B. dothidea* genome. Furthermore, using the CRISPR/Cas9 system, we edited the BdHsp20_1 gene that strongly responded to the DT treatment. As a result, the mycelial growth of the ΔBdHsp20 strain was 60.98% slower than that of the wild-type strain. Moreover, the disease severity of apple fruits inoculated with the ΔBdHsp20 strain was 97.43% lower than that inoculated with the wild-type strain. However, the BdHsp20 complemented strain, ΔBdHsp20_C, fully restored the growth and pathogenicity of *B. dothidea*, which showed that the BdHsp20 gene was closely related to the development and the virulence of the fungus *B. dothidea*.

In recent years, due to its importance in alleviating various biotic and abiotic stresses, the Hsp20 gene family was identified in many species. Our study identified 3–4 Hsp20 genes in six fungal species, such as *B. dothidea, L. theobromae, D. seriata, P. nodorum, Z. tritici*, and *V. mali*. Moreover, in a previous study, 1–5 Hsp20 genes were identified in 31 fungal species, including 19 *Aspergillus* species, four *Penicillium* species, three *Fusarium* species, three *Magnaporthe* species, as well as *Botrytis cinerea, Neurospora crassa*, and *Saccharomyces cerevisiae* (Wu et al., 2016). In *C. trogii*, there existed 14 Hsp20 genes (Wang et al., 2021b), which was more than those in the aforementioned fungal species. However, the number of Hsp20 genes in the fungi was much less than that in plant species such as apples (Yao et al., 2020), tomatoes (42) (Yu et al., 2016), potatoes (42) (Zhao et al., 2018), pumpkin (33) (Hu et al., 2021), grape (48) (Ji et al., 2019), African bermudagrass (41) (Cui et al., 2021), soybean (51) (Lopes-Caitar et al., 2013), barley (38) (Li and Liu, 2019), and bread wheat (163) (Muthusamy et al., 2017). All organisms continuously evolve under changing environmental conditions. During the natural selection process, they produced the genes that could produce proteins quickly in response to external stimuli, such as Hsp genes. Compared to fungi, plants are higher organisms with longer life cycles, which may have a more complicated biological process or suffer inevitable environmental stresses such as drought, salinity, high temperature, oxidative, and pathogen during growth and development processes. To better survive, plants needed to evolve more genes with similar structures and functions, such as Hsp20 genes, than fungi to respond to environmental stresses or deal with the more complicated metabolisms.

Our study showed that the four BdHsp20 genes contained a conserved α-crystallin domain (ACD)_sHsps-like domain with a variable N-terminal region and a short C-terminal extension. A previous study showed that the central ACD domain was the typical feature of the Hsp20 gene family (Kriehuber et al., 2010). The ACD participated in substrate interactions (Kirschner et al., 2000), the N terminus was involved in substrate binding (Basha et al., 2006), and the C-terminal extension was responsible for homo-oligomerization (Giese and Vierling, 2004). The three parts contributed to the function of sHsps as chaperone proteins (Lindner et al., 2000; Haslbeck et al., 2004).

In our study, each of the four BdHsp20 genes had no more than one intron, which was in line with the previous study, which revealed that most Hsp20 genes have no or only one intron. For example, 92.6% (38) of Hsp20 genes in apples (Yao et al., 2020), 93.8% (45) in grapes (Ji et al., 2019), and 89.6% (43) in potatoes (Zhao et al., 2018) have one intron or intronless. The intron density of annotated eukaryotic genomes varied by three orders of magnitude in the average number of introns per gene. For instance, there were 8.4 and 4.3 introns per gene in the *Homo sapiens* and *Arabidopsis thaliana* genomes, respectively, while only 0.0075 introns per gene in the *Encephalitozoon cuniculi* (Mourier and Jeffares, 2003). All eukaryotes evolved from a common ancestor. Some organisms lost many introns during evolution, whereas others gained many introns. These differences were subject to selection acting on introns depending on the organism's biology and the gene involved. The resulting consequence was that organisms that reproduce rapidly tend to have fewer introns than organisms with longer life cycles (Jeffares et al., 2006), which might explain the fewer introns in fungi than in plants. In addition, introns were the source of gene sequence variation (Jacob and Smith, 2017; Naro and Sette, 2017). The fewer introns in Hsp20 genes also revealed that the Hsp20 gene family was highly conserved.

Among the four BdHsp20 genes, BdHsp20_1 and BdHsp20_2 were paralogous gene pairs with 100% sequence identity, so they were the most closely related, which is consistent with the phylogenetic analysis.

Among the six fungal species, *B. dothidea, L. theobromae*, and *D. seriata* belonged to the family Botryosphaeriales. Therefore, Hsp20 from the three fungi showed a closer phylogenetic relationship than that in the other three fungi (*P. nodorum, Z. tritici*, and *V. mali*), which implied the Hsp20 gene and the species might have a similar evolution process. Furthermore, the collinear relationship is strongly related to the divergence time between species. Species with more collinearity have a shorter divergence time. Conversely, species with less collinearity have a longer divergence time (Xie et al., 2018). Our study revealed that the collinear gene pairs identified between *B. dothidea* and *L. theobromae*/*D. seriata* were not found in *B. dothidea* and *Z. tritici*/*V. mali*, which may indicate that these orthologous pairs formed after the divergence of the family Botryosphaeriales species (*B. dothidea, L. theobromae*, and *D. seriata*) and the other fungal species (*Z. tritici* and *V. mali*). In addition, some collinear pairs identified between *B. dothidea* and *L. theobromae* were also found in *B. dothidea* and *D. seriata*, indicating that these orthologous pairs might already exist before their divergence.

Our study proved that the BdHsp20 gene was closely associated with the growth and pathogenicity of *B. dothidea*. Previous studies showed *Ustilago maydis* Hsp20 gene was involved in the pathogenicity toward the host maize. Deleting the hsp20 gene decreases the severity of infection caused by the pathogen (Ghosh, 2014). In addition, Hsp20 was critical in maintaining the homeostasis of *Bacillus thuringiensis* during the production of spores and insecticidal crystal proteins (ICPs), and deletion of Hsp20 resulted in a decrease in both sporulation and ICPs production (Xie et al., 2019). Together with these previous studies, our present study demonstrated that the Hsp20 gene played an essential role in fungal growth and pathogenicity.

It is still noteworthy that DT significantly suppressed the *B. dothidea* growth, reduced the pathogenicity (Sun et al., 2022), and significantly upregulated the expression of the BdHsp20 gene. However, the BdHsp20 gene-edited strain significantly reduced the growth and pathogenicity of *B. dothidea*, which seems contradictory. We deduced that the Hsp20 possessed at least two functions for this inconsistent results. First, Hsp proteins are molecular chaperonins. They guarantee the correct tri-dimensional conformation of proteins during their synthesis or upon cellular stress, and they are also involved in eliminating incorrectly folded proteins if repair is impossible (Martine and Rebe, 2019). In this way, they protect the cell from various abiotic environmental stresses (Singh et al., 2014; Deng et al., 2020; Guo et al., 2020; Wang et al., 2021b). Then, Hsp20 participated in the fungal growth and pathogenicity (Ghosh, 2014; Xie et al., 2019). Therefore, we speculated that when *B. dothidea* was exposed to DT, the BdHsp20 gene was significantly upregulated immediately, which activated the chaperone function to relieve damage to the fungal cells. However, as the DT treatment time was prolonged, the expression of the BdHsp20 gene gradually decreased, indicating that the molecular chaperonin function weakened. By contrast, the function of regulating growth and pathogenicity gradually strengthened, resulting in decreased growth and pathogenicity.

## Conclusion

In conclusion, there were four BdHsp20 genes throughout the *B. dothidea* genome, each containing an conserved ACD. The segmental duplication event has contributed to the expansion of the BdHsp20 gene family. The purifying selection might have provided the primary impetus during the evolution of the BdHsp20 gene family. The BdHsp20 gene-edited strain reduced the mycelial growth and the pathogenicity, indicating BdHsp20 was involved in the growth and pathogenicity of *B. dothidea*. The present study would be helpful for further understanding the molecular mechanism by which DT inhibited the apple ring rot.

## Data availability statement

The original contributions presented in the study are included in the article/supplementary material, further inquiries can be directed to the corresponding authors.

## Author contributions

YH and YD contributed to the conception of the study and wrote and reviewed the manuscript. JLiu, JLi, and MS performed the experiment and collected the data. All authors contributed to the article and approved the submitted version.

## Funding

This work was supported by the National Natural Science Foundation of China (31471864), the Natural Science Foundation of Shandong Province (ZR2020MC143 and ZR2020MC136), the Agricultural Variety Improvement Project of Shandong Province 2020LZGC007, and the Qingdao Agricultural University High level Personnel Startup Fund China (6631115024).

## Conflict of interest

The authors declare that the research was conducted in the absence of any commercial or financial relationships that could be construed as a potential conflict of interest.

## Publisher's note

All claims expressed in this article are solely those of the authors and do not necessarily represent those of their affiliated organizations, or those of the publisher, the editors and the reviewers. Any product that may be evaluated in this article, or claim that may be made by its manufacturer, is not guaranteed or endorsed by the publisher.

## Supplementary material

Supplementary Table 1 The qRT-PCR primers used in the study.

Supplementary Table 2 Motif sequences of the Hsp20 gene family of *Botryosphaeria dothidea*.

Supplementary Table 3 Promoter prediction of BdHsp20 gene family by Promoter 2.0.

Supplementary Table 4 Promoter prediction of BdHsp20 gene family by Neural Network Promoter Prediction.

Supplementary Figure 1 Construction of BdHsp20 gene-edited and complement expression vector. **(A)** Schematic diagram of the Cas9/sgRNA vector pX458. **(B)** The Schematic illustration of the construction procession of the recombinant plasmid PX458-BdHsp20. **(C)** Schematic diagram of the complement vector PRI101. **(D)** The Schematic illustration of the construction procession of the recombinant plasmid PRI101-BdHsp20. The underlined sequence indicates restriction enzyme sites.

Supplementary Figure 2 The comparison of BdHsp20 in the wild-type strain and the ΔBdHsp20 strain. The alignment of the CDS of the BdHsp20 gene in wild-type and the ΔBdHsp20 strain **(A)**. The CDS and the coded protein of BdHsp20 in the wild-type strain **(B)** and the ΔBdHsp20 strain **(C)**. ATG and TGA marked in red indicate the start codon and the stop codon, respectively. The alignment of amino acid sequences of BdHsp20 protein in wild-type strain and the ΔBdHsp20 strain **(D)**. The underlined amino acid sequences consisted of the conserved alpha-crystallin domain (ACD). The tertiary structure model of the BdHsp20 protein in the wild-type strain **(E)** and the ΔBdHsp20 strain **(F)**.
