# Supplementary material for: The heat shock protein 20 gene editing suppresses mycelial growth of Botryosphaeria dothidea and decreases its pathogenicity to postharvest apple fruits
Source: Front Microbiol. 2022 Jul 27;13:930012. doi: 10.3389/fmicb.2022.930012 (PMC9363843; doi:10.3389/fmicb.2022.930012)
Supplement: Supplementary Table 1 — The qRT-PCR primers used in the study. [file Data_Sheet_1.docx]

## Supplementary Table S1-S4

### Supplementary Table S1

| Supplementary Table S1 The qRT-PCR primers used in the study | | |
| --- | --- | --- |
| **Gene** | **Forward primerSequence** | **Reverse primerSequence** |
| BOTSDO12492 | 5'- GCGCTTCACTCAGGAGTTCT -3' | 5'- TTCTTCTCCGCAACGTCGAA-3' |
| BOTSDO02788 | 5'- TTCGACGTTGCGGAGAAGAA -3' | 5'- CAGAGTGTTGGAGTCGGACC-3' |
| BOTSDO08183 | 5'-GAGAGCGCGTACGTAATCCA-3' | 5'-CCTGATCTTGCGCTCAAACG -3' |
| BOTSDO09929 | 5'-CCACTACCAGCGGACATTCA-3' | 5'-CTCACGACCTTGGGAACCAG -3' |
| actin | 5'-GTTCAGACCGCCCTTTGCT-3' | 5'-AGCCTTGCGACGGAACATA-3' |

### Supplementary Table 2

| Supplementary Table S2 Motif sequences of the Hsp20 gene family of *Botryosphaeria dothidea* | | |  |
| --- | --- | --- | --- |
| Motif | Width | Sequence | |
| 1 | 50 | MSMFPRFTQEFSPLFRLLDDYDRGTQSIRSFTPKFDVAEKKEAYELHGEL | |
| 2 | 29 | RSFSFPGRVDQDNIKASLKDGILRITVPK | |
| 3 | 48 | IAQKDINIEWSDSNTLTISGRTEHHSERGERPQGFIEGEEHGYQKPSV | |
| 4 | 29 | AKTNENKEVGKPNDEVKFWVSERSVGEFH | |
| 5 | 11 | AQAPKARRINI | |
| 6 | 10 | CPHHHRRGVC | |
| 7 | 7 | QEKSKEV | |
| 8 | 11 | DVRETESAFVJ | |
| 9 | 6 | HAEAFQ | |
| 10 | 6 | IEKVDJ | |

### Supplementary Table S3

| Supplementary Table S3 Promoter prediction of BdHsp20 gene family by Promoter2.0 | | | |
| --- | --- | --- | --- |
| Gene | Position | Score | Likelihood |
| BdHsp20_1 | 1600 | 0.568 | Marginal prediction |
| BdHsp20_2 | 1600 | 0.568 | Marginal prediction |
| BdHsp20_3 | 1100 | 0.593 | Marginal prediction |
|  | 1500 | 0.528 | Marginal prediction |
| BdHsp20_4 | no |  |  |

### Supplementary Table S4

| Supplementary Table S4 Promoter prediction of BdHsp20 gene family by Neural Network Promoter Prediction | | | | |
| --- | --- | --- | --- | --- |
| Gene | Start | End | Score | Promoter_Sequence |
| BdHsp20_1 | 799 | 849 | 0.87 | CGGAGCATTATCAAAAAGGGGAAAAGGACAAGAAGAGGCTTGCTAGATCA |
|  | 1392 | 1442 | 0.9 | AGCGCCATCGAGTATATGCTCCGCAAAGGCCAGAGACAACTAGAGATCTA |
|  | 1814 | 1864 | 1 | AGAACTGCGTGTATATAATGGTGCCTGATGGACGGCATTCAGCTCGCCAG |
| BdHsp20_2 | 799 | 849 | 0.87 | CGGAGCATTATCAAAAAGGGGAAAAGGACAAGAAGAGGCTTGCTAGATCA |
|  | 1392 | 1442 | 0.9 | AGCGCCATCGAGTATATGCTCCGCAAAGGCCAGAGACAACTAGAGATCTA |
|  | 1814 | 1864 | 1 | AGAACTGCGTGTATATAATGGTGCCTGATGGACGGCATTCAGCTCGCCAG |
| BdHsp20_3 | 1119 | 1169 | 0.95 | TAAGTTGACCTCAAAAGGCCGGAATACGGTGAAGCTCAAGATTGGCGGTT |
|  | 1366 | 1416 | 0.82 | TTTCTTTGGATAGAAGCGACGGCGAAAGGTAGGGCCGGTCAGAATCACGT |
|  | 1913 | 1963 | 0.99 | ACCTCGTCTTCTATTTAAGCCGCCGCGCAGCCCCAGGAACAGGCCCATCA |
| BdHsp20_4 | 127 | 177 | 0.93 | CAAGGGAGGAGAAAAAACAGGGCTTACGGGCTCCAGCAGCAGCGGGCCGT |
|  | 1620 | 1670 | 0.96 | TCTCACGCCCTATAAGGACCACCGTCGTTTGGTCCAAATCACGGATTTGC |
|  | 1792 | 1842 | 0.83 | CCAAAACGGCAATAAAACTAGCACCACCGGACCTCTCCCCTCCGCCCACA |
|  | 1909 | 1959 | 0.89 | CCCTCCCACACAAAAACACCCCCACGACCATCAAAACGGCGGCCATCCTC |
|  | 1928 | 1978 | 0.99 | CCCCACGACCATCAAAACGGCGGCCATCCTCCCCCTCCCCGTCGTCCCGG |
