# Supplementary figures and images for: The heat shock protein 20 gene editing suppresses mycelial growth of Botryosphaeria dothidea and decreases its pathogenicity to postharvest apple fruits
Source: Front Microbiol. 2022 Jul 27;13:930012. doi: 10.3389/fmicb.2022.930012 (PMC9363843; doi:10.3389/fmicb.2022.930012)

Supplementary Figure S1

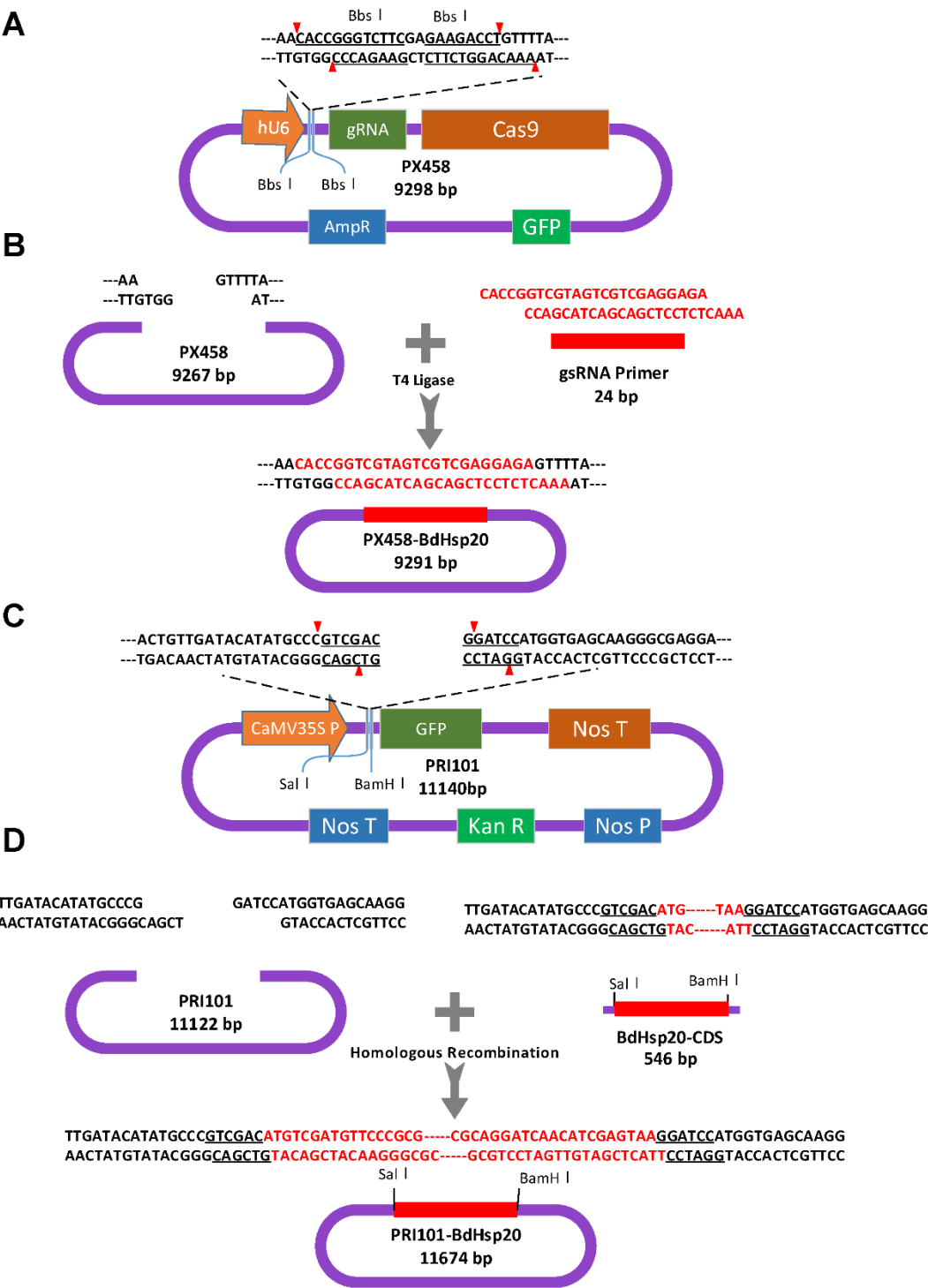

Supplement: Supplementary Figure 1 — Construction of BdHsp20 gene-edited and complement expression vector. (A) Schematic diagram of the Cas9/sgRNA vector pX458. (B) The Schematic illustration of the construction procession of the recombinant plasmid PX458-BdHsp20. (C) Schematic diagram of the complement vector PRI101. (D) The Schematic illustration of the construction procession of the recombinant plasmid PRI101-BdHsp20. The underlined sequence indicates restriction enzyme sites. [file Image_1.pdf]
