# Supplementary material for: The heat shock protein 20 gene editing suppresses mycelial growth of Botryosphaeria dothidea and decreases its pathogenicity to postharvest apple fruits
Source: Front Microbiol. 2022 Jul 27;13:930012. doi: 10.3389/fmicb.2022.930012 (PMC9363843; doi:10.3389/fmicb.2022.930012)

Supplementary Figure S2

A

```
gRNA      ----------------------------------------------------------------------------------------------------
WT          1 ATGTCGATGTTCCCGCGCTTCACTCAGGAGTTCTCTCCTCTCTTCCGTCTCCTCGACGACTACGACCGCGGAACTCAAAGCATTC  85
ΔBdHsp20    1 ATGTCGATGTTCCCGCGCTTCACTCAGGAGTTCTCTCCTCTCTTCCGTCTCCTCGACGACTACGACCGCGGAACTCAAAGCATTC  85

gRNA        1 ----------------------------------------------------------ACTCCCTGGCATTGCTCAGA----  20
WT         86 GGTCTTTCACTCCCAAGTTCGACGTTGCGGAGAAGAAGGAAGCCTACGAGCTTCAGGGCGAACTCCCTGGCATTGCTCAGAAGGA 170
ΔBdHsp20   86 GGTCTTTCACTCCCAAGTTCGACGTTGCGGAGAAGAAGGAAGCCTACGAGCTTCAC-GCGAACTCCCTGGCATTGCCAGAAGGA 169

gRNA      ----------------------------------------------------------------------------------------------------
WT        171 CATCAACATCGAATGGTCCGACTCCAACACTCTGACCATCTCAGGTCGCACCGAGCACCACTCCGAGCGTGGCGAGCGCCCTCAG 255
ΔBdHsp20  170 CATCAACATCGAATGGTCCGACTCCAACACTCTGACCATCTCAGGTCGCACCGAGCACCACTCCGAGCGTGGCGAGCGCCCTCAG 254

gRNA      ----------------------------------------------------------------------------------------------------
WT        256 GGTTTCATTGAGGGTGAGGAGCATGGCTACCAGAAGCCCTCCGTCGAGGAAGAGCCCCAGGAGAAGAGCAAGGAGGTGGCCAAGA 340
ΔBdHsp20  255 GGTTTCATTGAGGGTGAGGAGCATGGCTACCAGAAGCCCTCCGTCGAGGAAGAGCCCCAGGAGAAGAGCAAGGAGGTGGCCAAGA 339

gRNA      ----------------------------------------------------------------------------------------------------
WT        341 CCAACGAAAACAAGGAGGTCGGCAAGCCTAACGATGAAGTCAAGTTCTGGGTGAGCGAGCGCAGCGTTGGCGAATTCCACCGCTC 425
ΔBdHsp20  340 CCAACGAAAACAAGGAGGTCGGCAAGCCTAACGATGAAGTCAAGTTCTGGGTGAGCGAGCGCAGCGTTGGCGAATTCCACCGCTC 424

gRNA      ----------------------------------------------------------------------------------------------------
WT        426 GTTCAGCTTCCCCGGCCGCGTCGATCAGGACAATGTCAAGGCTAGCCTGAAGGACGGCATCCTCACCATCACCGTCCCCAAGGCC 510
ΔBdHsp20  425 GTTCAGCTTCCCCGGCCGCGTCGATCAGGACAATGTCAAGGCTAGCCTGAAGGACGGCATCCTCACCATCACCGTCCCCAAGGCC 509

gRNA      ------------------------------------
WT        511 CAGGCCCCCAAGGCTCGCAGGATCAACATCGAGTAA 546
ΔBdHsp20  510 CAGGCCCCCAAGGCTCGCAGGATCAACATCGAGTAA 545
```

B

```
Translation of WT(1-546)
Universal code
Total amino acid number: 181, MW=20713
Max ORF starts at AA pos 1(may be DNA pos 1) for 181 AA(543 bases), MW=20713
                10        20        30        40        50        60        70        80        90
1         ATGTCGATGTTCCCGCGCTTCACTCAGGAGTTCTCTCCTCTCTTCCGTCTCCTCGACGACTACGACCGCGGAACTCAAAGCATTCGGTCTTTC
1          M  S  M  F  P  R  F  T  Q  E  F  S  P  L  F  R  L  L  D  D  Y  D  R  G  T  Q  S  I  R  S  F

                103       113       123       133       143       153       163       173       183
94        ACTCCCAAGTTCGACGTTGCGGAGAAGAAGGAAGCCTACGAGCTTCACGGCGAACTCCCTGGCATTGCTCAGAAGGACATCAACATCGAATGG
32         T  P  K  F  D  V  A  E  K  K  E  A  Y  E  L  H  G  E  L  P  G  I  A  Q  K  D  I  N  I  E  W

                196       206       216       226       236       246       256       266       276
187       TCCGACTCCAACACTCTGACCATCTCAGGTCGCACCGAGCACCACTCCGAGCGTGGCGAGCGCCCTCAGGGTTTCATTGAGGGTGAGGAGCAT
63         S  D  S  N  T  L  T  I  S  G  R  T  E  H  H  S  E  R  G  E  R  P  Q  G  F  I  E  G  E  E  H

                289       299       309       319       329       339       349       359       369
280       GGCTACCAGAAGCCCTCCGTCGAGGAAGAGCCCCAGGAGAAGAGCAAGGAGGTGGCCAAGACCAACGAAAACAAGGAGGTCGGCAAGCCTAAC
94         G  Y  Q  K  P  S  V  E  E  E  P  Q  E  K  S  K  E  V  A  K  T  N  E  N  K  E  V  G  K  P  N

                382       392       402       412       422       432       442       452       462
373       GATGAAGTCAAGTTCTGGGTGAGCGAGCGCAGCGTTGGCGAATTCCACCGCTCGTTCAGCTTCCCCGGCCGCGTCGATCAGGACAATGTCAAG
125        D  E  V  K  F  W  V  S  E  R  S  V  G  E  F  H  R  S  F  S  F  P  G  R  V  D  Q  D  N  V  K

                475       485       495       505       515       525       535       545
466       GCTAGCCTGAAGGACGGCATCCTCACCATCACCGTCCCCAAGGCCCAGGCCCCCAAGGCTCGCAGGATCAACATCGAGTAA
156        A  S  L  K  D  G  I  L  T  I  T  V  P  K  A  Q  A  P  K  A  R  R  I  N  I  E  *
```

C

```
Translation of ΔHsp20(1-545)
Universal code
Total amino acid number: 178, MW=19333
Max ORF starts at AA pos 1(may be DNA pos 1) for 67 AA(201 bases), MW=7686
                10        20        30        40        50        60        70        80        90
1         ATGTCGATGTTCCCGCGCTTCACTCAGGAGTTCTCTCCTCTCTTCCGTCTCCTCGACGACTACGACCGCGGAACTCAAAGCATTCGGTCTTTC
1          M  S  M  F  P  R  F  T  Q  E  F  S  P  L  F  R  L  L  D  D  Y  D  R  G  T  Q  S  I  R  S  F

                103       113       123       133       143       153       163       173       183
94        ACTCCCAAGTTCGACGTTGCGGAGAAGAAGGAAGCCTACGAGCTTCACGCGAACTCCCTGGCATTGCCCAGAAGGACATCAACATCGAATGGT
32         T  P  K  F  D  V  A  E  K  K  E  A  Y  E  L  H  A  N  S  L  A  L  P  R  R  T  S  T  S  N  G

                196       206       216       226       236       246       256       266       276
187       CCGACTCCAACACTCTGACCATCTCAGGTCGCACCGAGCACCACTCCGAGCGTGGCGAGCGCCCTCAGGGTTTCATTGAGGGTGAGGAGCATG
63         P  T  P  T  L  T  I  S  G  R  T  E  H  H  S  E  R  G  E  R  P  Q  G  F  I  E  G  E  E  H  A

                289       299       309       319       329       339       349       359       369
280       GCTACCAGAAGCCCTCCGTCGAGGAAGAGCCCCAGGAGAAGAGCAAGGAGGTGGCCAAGACCAACGAAAACAAGGAGGTCGGCAAGCCTAACG
94

                382       392       402       412       422       432       442       452       462
373       ATGAAGTCAAGTTCTGGGTGAGCGAGCGCAGCGTTGGCGAATTCCACCGCTCGTTCAGCTTCCCCGGCCGCGTCGATCAGGACAATGTCAAGG
125

                475       485       495       505       515       525       535       545
466       CTAGCCTGAAGGACGGCATCCTCACCATCACCGTCCCCAAGGCCCAGGCCCCCAAGGCTCGCAGGATCAACATCGAGTAA
156
```

D

```
WT          1 MSMFPRFTQEFSPLFRLLDDYDRGTQSIRSFTPKFDVAEKKEAYELHGELPGIAQKDINIEWSDSNTLTISGRTEHHSERGERPQ  85
ΔBdHsp20    1 MSMFPRFTQEFSPLFRLLDDYDRGTQSIRSFTPKFDVAEKKEAYELH----------------ANSLALPRRTS--TSNGPTPT  66

WT         86 GFIEGEEHGYQKPSVEEEPQEKSKEVAKTNENKEVGKPNDEVKFWVSERSVGEFHRSFSFPGRVDQDNVKASLKDGILTITVPKA 170
ΔBdHsp20   67 L---------------------------------------------------------------------------------- 67

WT        171 QAPKARRINIE                                                                          181
ΔBdHsp20      -----------
```

E

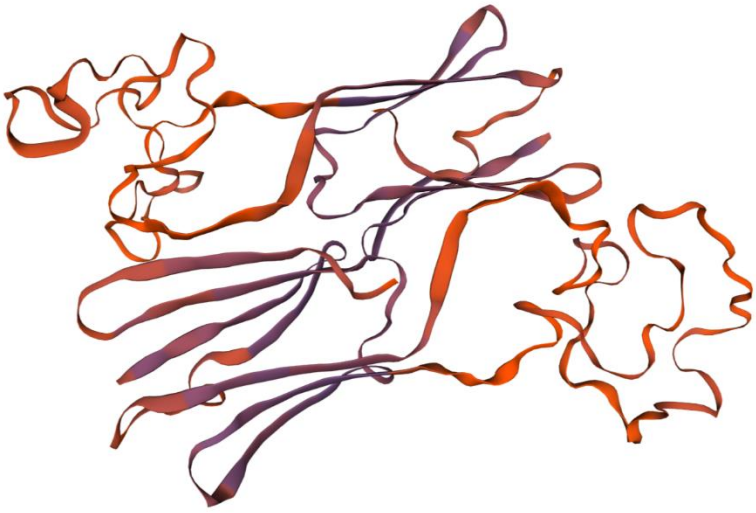

F

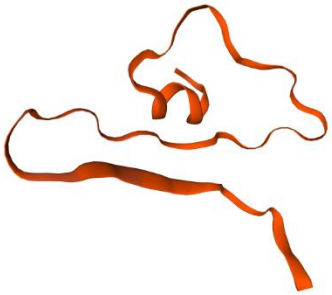

Supplement: Supplementary Figure 2 — The comparison of BdHsp20 in the wild-type strain and the ΔBdHsp20 strain. The alignment of the CDS of the BdHsp20 gene in wild-type and the ΔBdHsp20 strain (A). The CDS and the coded protein of BdHsp20 in the wild-type strain (B) and the ΔBdHsp20 strain (C). ATG and TGA marked in red indicate the start codon and the stop codon, respectively. The alignment of amino acid sequences of BdHsp20 protein in wild-type strain and the ΔBdHsp20 strain (D). The underlined amino acid sequences consisted of the conserved alpha-crystallin domain (ACD). The tertiary structure model of the BdHsp20 protein in the wild-type strain (E) and the ΔBdHsp20 strain (F). [file Image_2.pdf]
